# Supplementary material for: The cichlid oral and pharyngeal jaws are evolutionarily and genetically coupled
Source: Nat Commun. 2021 Sep 16;12:5477. doi: 10.1038/s41467-021-25755-5 (PMC8445992; doi:10.1038/s41467-021-25755-5)
Supplement: Supplementary file 1 — Supplementary Information [file 41467_2021_25755_MOESM1_ESM.pdf]

Supplementary Information for

The cichlid oral and pharyngeal jaws are evolutionarily and genetically coupled

Andrew J. Conith[1*], R. Craig Albertson[1*]

[1] Biology Department, University of Massachusetts Amherst, Amherst, MA, 01003

*Corresponding authors

AJC: ajconith@bio.umass.edu

RCA: albertson@bio.umass.edu

Contents

Part I. Additional Methods

Part II. Supplementary Figures

Supplementary Figures 1 to 7

Part III. Additional Citations

Part IV. Citations for diet assignments in Supplementary Data 2

Part I. Additional Methods

Fish Husbandry

Cichlids were reared in 40-gallon glass aquaria on a 14hr light/10hr dark daily cycle in the Albertson lab at the University of Massachusetts Amherst. Animals were kept in a recirculating system and water was automatically regulated to keep temperature, salinity, and pH consistent. Animals were fed a mixture of algae (Spirulina) and egg yolk flake food, thus limiting environmental variation that could contribute to differences in the craniofacial shape of our hybrid populations. Over time, smaller families were combined together and larger families were split up to gain an optimal number of individuals per tank that permitted growth while limiting dominance behaviors common to lake Malawi cichlids. Hybrid animals were euthanized in MS-222 at ~5 months and fin clipped for RAD-seq before fixation in 4% paraformaldehyde in preparation for μCT scanning. All experiments involving animals was approved by the University of Massachusetts institutional animal care and use committee (IACUC).

Additional 3D Geometric Morphometric Analysis

To characterize oral and pharyngeal jaw shape across in all different groups we followed the same landmarking scheme in each (Supplementary Fig. 1; Supplementary Data 16). We first performed Procrustes superimposition to remove effects of scale, translation and rotation using the `gpagen` *geomorph* function in R [1,2]. Following Procrustes superimposition, if multiple individuals were digitized from a single species in the macroevolutionary (across African cichlids) and microevolutionary (across *Tropheops* sp.) components of this study, we calculated a species mean landmark configuration. We then conducted a Procrustes ANOVA between the centroid size and shape of each structure in R [2] using the `procD.lm` *geomorph* function, or the `procD.pgls` *geomorph* function when assessing among species, and found a significant effect of allometry in

almost all cases (Supplementary Data 17). To remove the allometric component of shape variation, we extracted the landmark residuals from this Procrustes ANOVA model to obtain landmark data sets for all four traits for use in subsequent analyses.

All structures were landmarked for both left and right sides separated by their midlines, allowing us to assess object symmetry and remove the effects of developmental noise. First, the landmarks from one side are reflected onto the other and undergo Procrustes superimposition. The variation among the reflected and original landmark configurations for all comprises the symmetric component of shape variation [3]. The degree of symmetry was then statistically evaluated using Procrustes ANOVA using residual randomization permutation procedures [4]. All geometric morphometric datasets underwent symmetric corrections (Supplementary Data 18).

RNA Extraction and Quantitative PCR

We dissected tissues from three species of cichlid to extract RNA and perform quantitative PCR (qPCR): *Labeotropheus fuelleborni* (LF, n = 8), *Maylandia callainos* (MC, n = 8), and *Tropheops kumara* (TK, n = 6). We dissected lower oral jaw and lower pharyngeal jaw tissues, and took a caudal fin clip to act as a control tissue. All tissues were placed into TRIzol Reagent (Ambion Life Technologies) to limit RNA degradation and homogenized using the Bullet Blender Storm Tissue Homogenizer and stainless steel UFO beads (Next Advance, Averill Park, NY, USA). RNA was isolated from homogenized tissues via a phenol/chloroform extraction technique and ethanol precipitation. We removed any genomic DNA (gDNA) from the samples by degrading the gDNA using a DNase enzyme (Invitrogen). We quantified the amount of RNA in the samples spectrophotometrically (NanoDrop 2000, Thermo Scientific) and standardized across all samples to 100ng/μL. We reverse transcribed RNA to cDNA using a High Capacity cDNA Reverse Transcription Kit (Ambion Life Technologies).

We used Primer3 software (http://bioinfo.ut.ee/primer3/) to design cichlid-specific primers for *dym*, *notch1a*, and *smad7* (dym-LG7: forward, 5'-GCACAGTTTCGATGTCTCCA-3', reverse, 5'-GCACAGGCAAGAGTTGATGA-3'; *notch1a*-LG7: forward, 5'-CAGATGCGAGCAGGACATAA-3', reverse, 5'-ACAGGTGCCACCATTAAAGC-3'; *smad7*-LG7: forward, 5'-CTCTCCCCATGCTGCTCTAC-3', reverse, 5'-CATGGGATAGCGGGAATATG-3'). For our reference gene, we used a previously published sequence for *β-actin* [5], as this primer pair was known to have high amplification efficiency (*β-actin*-LG4: forward, 5'-GTATGTGCAAGGCCGGATT-3', reverse, 5'-TTCTGACCCATACCCACCAT-3').

We confirmed the presence of cDNA in each sample by performing a PCR with *β-actin* and running on an electrophoresis gel. We also used this method to confirm our newly designed primers would amplify in our samples.

We assessed primer efficiencies using the standard curve method with concentrations of 0.8ng/μL, 4ng/μL, 20ng/μL, and 100ng/μL, and found all primers exhibited >90% efficiency (Supplementary Data 13; *dym* 92.92%, *notch1a* 99.89%, *smad7* 102.40%, *β-actin* 96.33%). We used qPCR to assess levels of gene expression using the SYBR Green chemistry method (Power SYBR Green Master Mix Applied Biosystems), and calculated relative expression using the $2^{-\Delta\Delta CT}$ technique using β-actin as the control gene, and caudal fin as the control tissue. We used the `pcr_analyze` function in the R package *pcr* to calculate all relative expression values [6].

Part II. Supplementary Figures

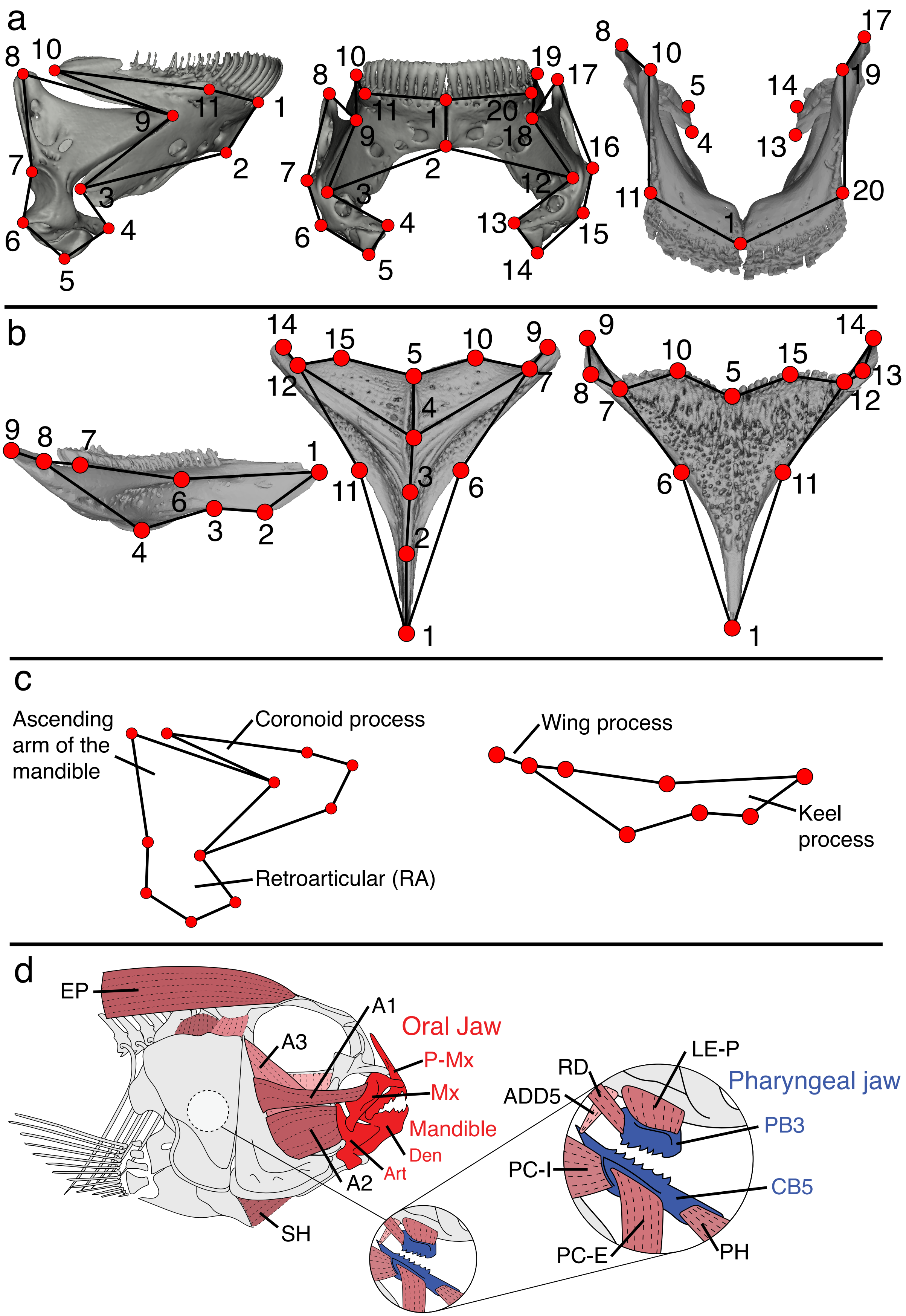

Supplementary Figure 1. Oral and pharyngeal jaw symmetric landmark configurations. See Supplementary Data 16 for full descriptions of landmark placement. a, Lower oral jaw μCT scans in, from left to right, lateral view, anterior view, dorsal view. b, Lower pharyngeal jaw μCT scans in, from left to right, lateral view, anterior view, dorsal view. Closed red circles indicate positions landmarks were place on each jaw. c, Major processes of each jaw in the lower oral (left) and lower pharyngeal jaw (right). d, Cichlid craniofacial schematic depicting major oral (red) and pharyngeal jaw (blue) bones, alongside major muscles involved in feeding and powering the jaws. Anatomical naming convention follows Liem [7]. Bones, Oral Jaw (red text): P-Mx, Pre-maxillia; Mx, Maxilla; Mandible (Lower oral jaw) comprises of Den, Dentary, and Art, Articular; Pharyngeal jaw (blue text): PB3, Third pharyngobranchial (upper pharyngeal jaw); CB5, Fifth ceratobranchial (Lower pharyngeal jaw). Muscles: A1, Adductor mandibulae A1; A2, Adductor mandibulae A2; A3, Adductor mandibulae A3; ADD5, Fifth adductor; EP, Epaxial; LE-P, Levator externus/posterior; PH, Pharyngohyoideus; PC-E, Pharyngoclithralis externus; PC-I, Pharyngoclithralis internus; RD, Retractor dorsalis; SH, Sternohyoideus. Scan images and line drawings were produced by the authors using MeshLab (v2019-12) and Adobe Illustrator CC (v22.0.1).

a

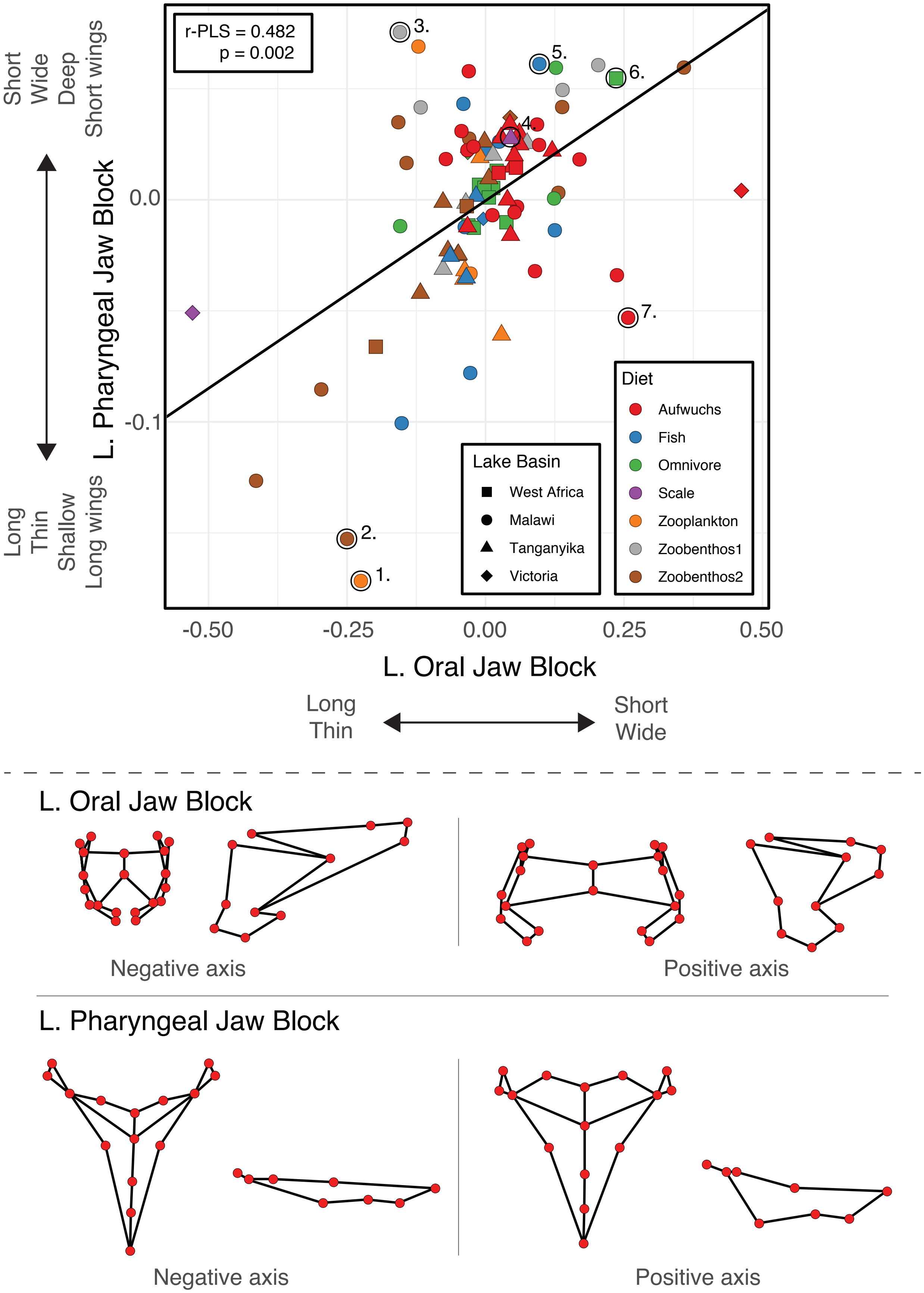
r-PLS = 0.482
p = 0.002
Short
Wide
Deep
Short wings
Long
Thin
Shallow
Long wings
L. Pharyngeal Jaw Block
0.0
-0.1
3.
5.
6.
4.
7.
2.
1.
Lake Basin
West Africa
Malawi
Tanganyika
Victoria
Diet
Aufwuchs
Fish
Omnivore
Scale
Zooplankton
Zoobenthos1
Zoobenthos2
-0.50
-0.25
0.00
0.25
0.50
L. Oral Jaw Block
Long
Thin
Short
Wide
L. Oral Jaw Block
Negative axis
Positive axis
L. Pharyngeal Jaw Block
Negative axis
Positive axis

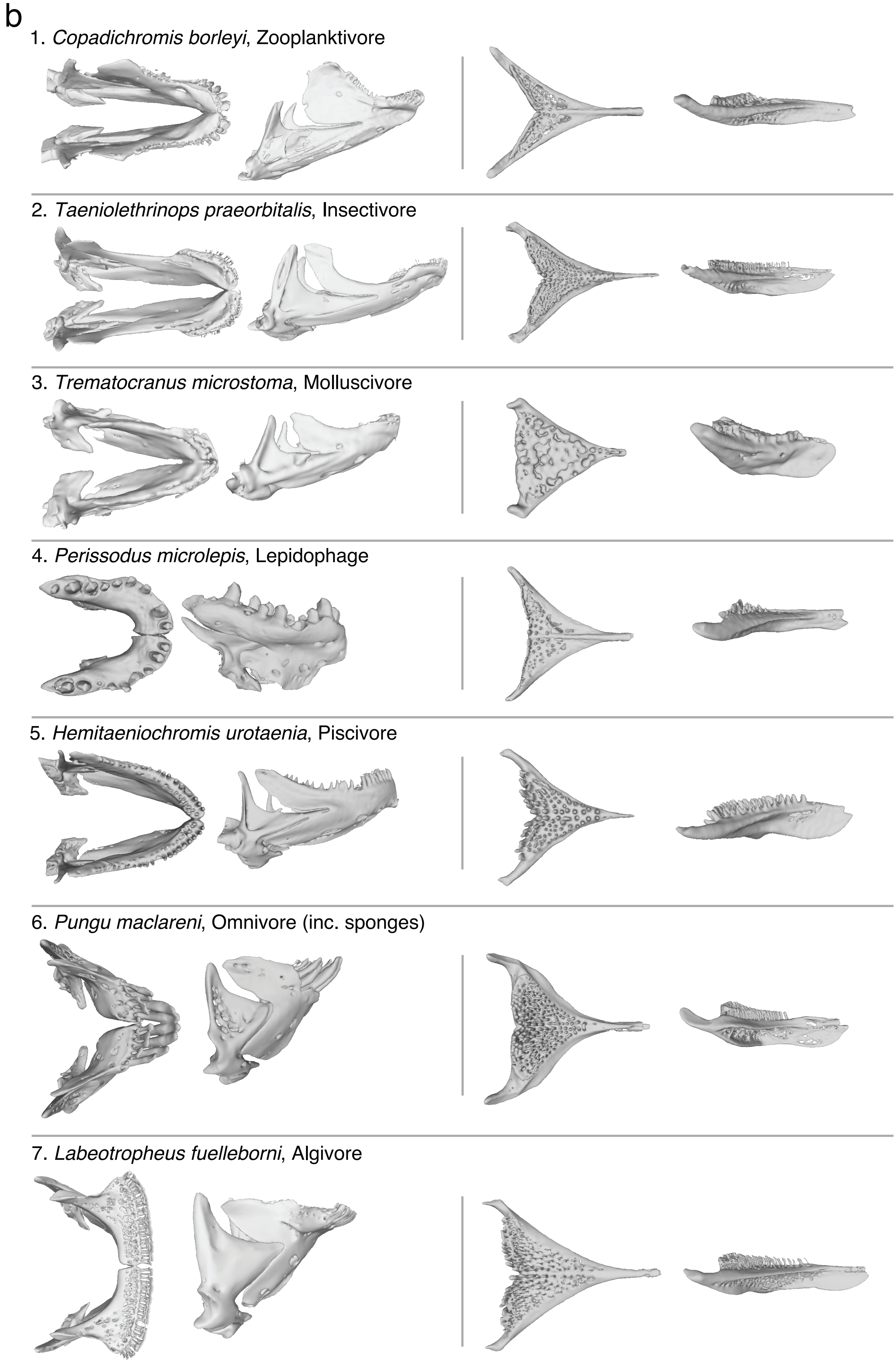
b
1. *Copadichromis borleyi*, Zooplanktivore
2. *Taeniolethrinops praeorbitalis*, Insectivore
3. *Trematocranus microstoma*, Molluscivore
4. *Perissodus microlepis*, Lepidophage
5. *Hemitaeniochromis urotaenia*, Piscivore
6. *Pungu maclareni*, Omnivore (inc. sponges)
7. *Labeotropheus fuelleborni*, Algivore

Supplementary Figure 2. Phylogenetically corrected two-block partial least squares analysis to assess macroevolutionary associations between lower oral and pharyngeal jaws. a, Two-block partial least squares with diet assignment mapped to each taxon. Diet information was obtained from the literature and citations for each assignment can be found in Supplementary Data 2 and Part IV of the supplementary text. Highlighted taxa with number assignments reflect cichlids that stray further from the best-fit line and/or exhibit more specialized morphologies. Wireframes depict representative jaw morphologies present at the extremes of each axis. b, Lower oral jaws and lower pharyngeal jaws of taxa highlighted in the two-block partial least squares analysis to better-illustrate associations between jaws. Diets for each taxon are also listed, references for which can be found in Supplementary Data 2 and Part IV of the supplementary text. Source data are provided as a Source Data file. Scan images were produced by the authors using MeshLab (v2019-12).

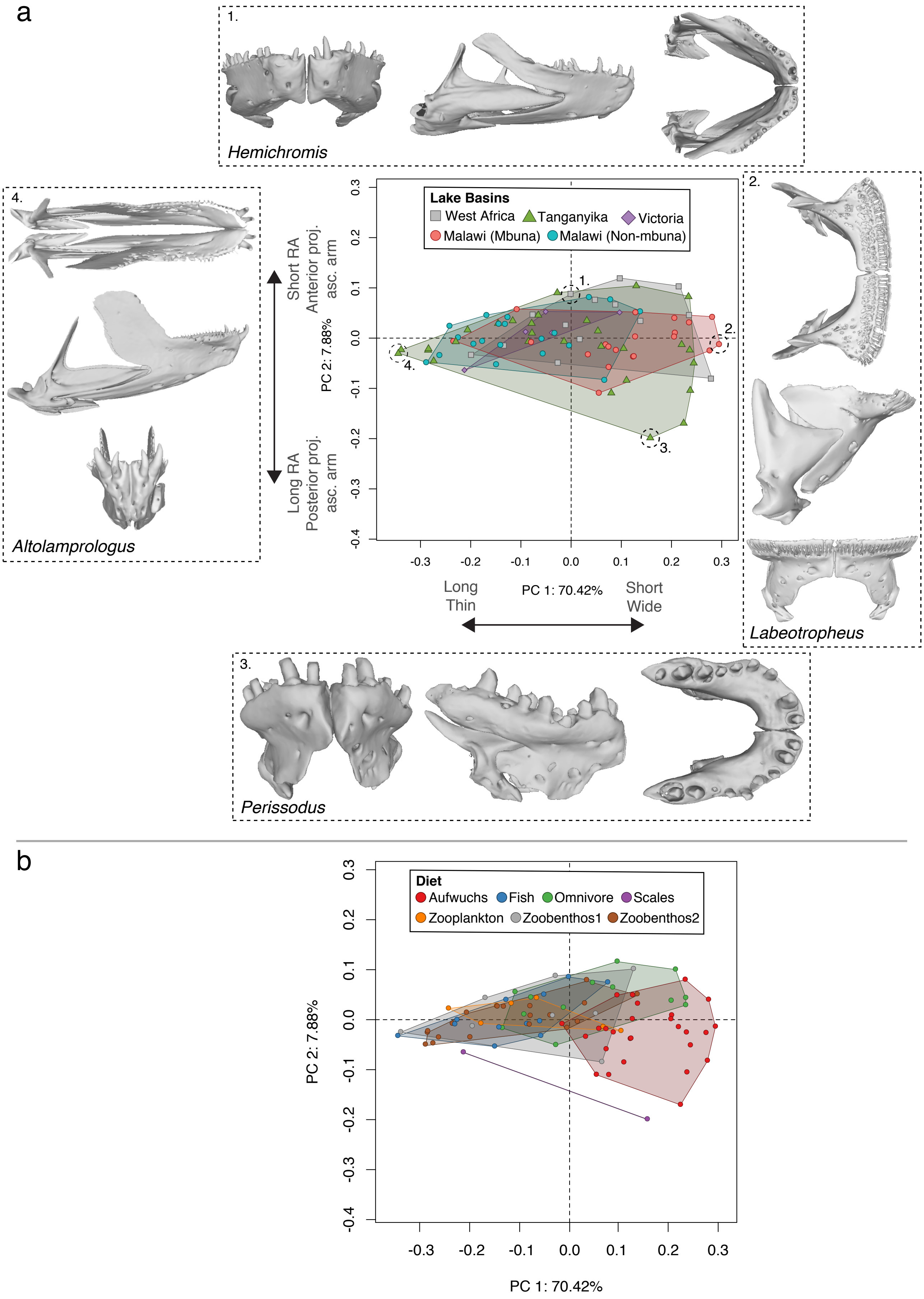
a
1.
Hemichromis
4.
Altolamprologus
2.
Labeotropheus
3.
Perissodus
Lake Basins
West Africa
Tanganyika
Victoria
Malawi (Mbuna)
Malawi (Non-mbuna)
Short RA
Anterior proj.
asc. arm
Long RA
Posterior proj.
asc. arm
PC 2: 7.88%
PC 1: 70.42%
Long
Thin
Short
Wide
0.3
0.2
0.1
0.0
-0.1
-0.2
-0.3
-0.4
-0.3
-0.2
-0.1
0.0
0.1
0.2
0.3
b
Diet
Aufwuchs
Fish
Omnivore
Scales
Zooplankton
Zoobenthos1
Zoobenthos2
PC 2: 7.88%
PC 1: 70.42%

c

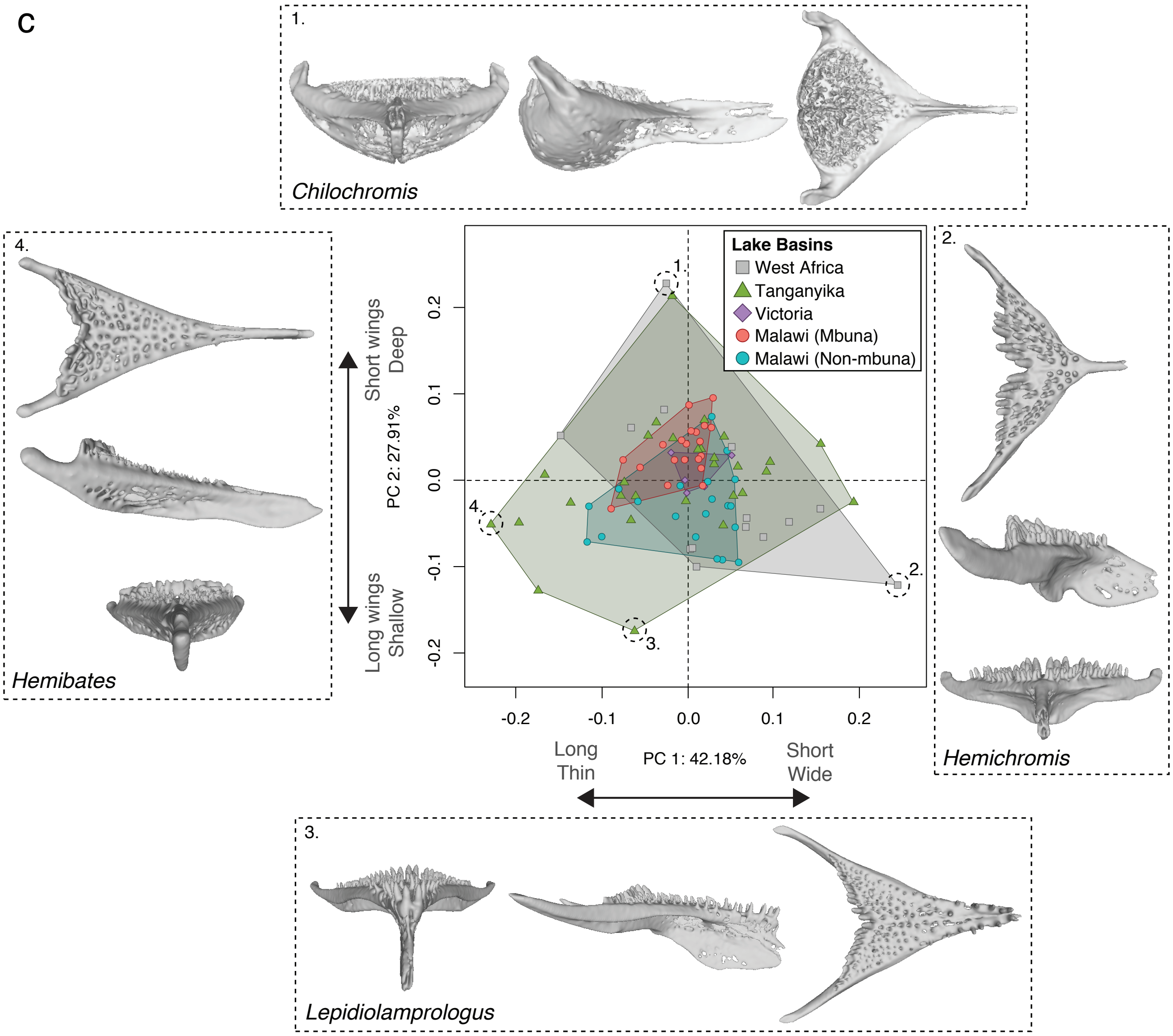

1.
Chilochromis
2.
Hemichromis
3.
Lepidiolamprologus
4.
Hemibates
Lake Basins
West Africa
Tanganyika
Victoria
Malawi (Mbuna)
Malawi (Non-mbuna)
PC 2: 27.91%
Short wings
Deep
Long wings
Shallow
PC 1: 42.18%
Long
Thin
Short
Wide
0.2
0.1
0.0
-0.1
-0.2

d

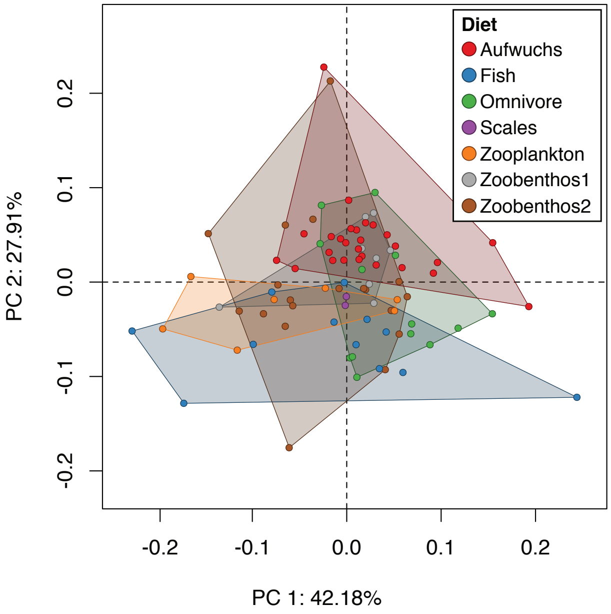

Diet
Aufwuchs
Fish
Omnivore
Scales
Zooplankton
Zoobenthos1
Zoobenthos2
PC 2: 27.91%
PC 1: 42.18%
0.2
0.1
0.0
-0.1
-0.2

Supplementary Figure 3. Principal components analysis of African cichlid jaw landmark data from the macroevolutionary component of the study. a, Lower oral jaw morphospace with convex hulls depicting the boundaries of shape space for each lake and or clade. Lower oral jaw scans reflect jaw morphologies of a specific taxon present at the extremes of each axis, and their associated number can identify their position in morphospace. b, Same morphospace as above with convex hulls now depicting the boundaries of shape space for each diet assignment. c, Lower pharyngeal jaw morphospace with convex hulls depicting the boundaries of shape space for each lake and or clade. Lower pharyngeal jaw scans reflect jaw morphologies of a specific taxon present at the extremes of each axis, and their associated number can identify their position in morphospace. d, Same morphospace as above with convex hulls now depicting the boundaries of shape space for each diet assignment. Diets for each taxon are also listed, references for which can be found in Supplementary Data 2 and Part IV of the supplementary text. Source data are provided as a Source Data file. Scan images were produced by the authors using MeshLab (v2019-12).

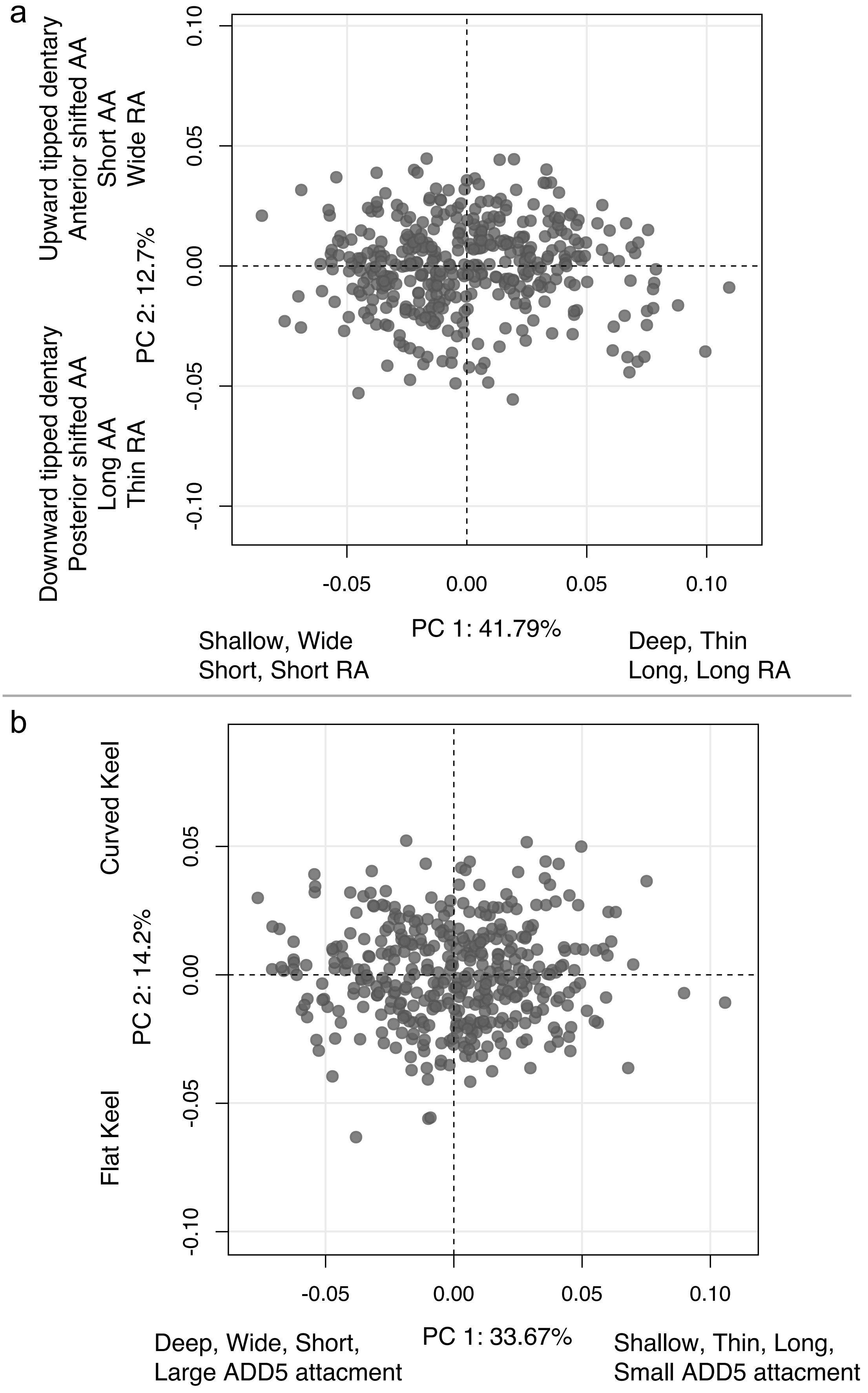
a
Upward tipped dentary
Anterior shifted AA
Short AA
Wide RA
Downward tipped dentary
Posterior shifted AA
Long AA
Thin RA
PC 2: 12.7%
0.10
0.05
0.00
-0.05
-0.10
-0.05
0.00
0.05
0.10
PC 1: 41.79%
Shallow, Wide
Short, Short RA
Deep, Thin
Long, Long RA
b
Curved Keel
Flat Keel
PC 2: 14.2%
0.05
0.00
-0.05
-0.10
-0.05
0.00
0.05
0.10
PC 1: 33.67%
Deep, Wide, Short,
Large ADD5 attacment
Shallow, Thin, Long,
Small ADD5 attacment

Supplementary Figure 4. Jaw shape morphospaces for all F_5 hybrids in this study. a, Principal component (PC) scores from the first two axes are plotted for the lower oral jaw landmark configurations. b, PC scores from the first two axes are plotted for the lower pharyngeal jaw landmark configurations. The percentage of variation explained by each axis is also presented, alongside anatomical descriptions of how shape changes across the axes. Source data are provided as a Source Data file.

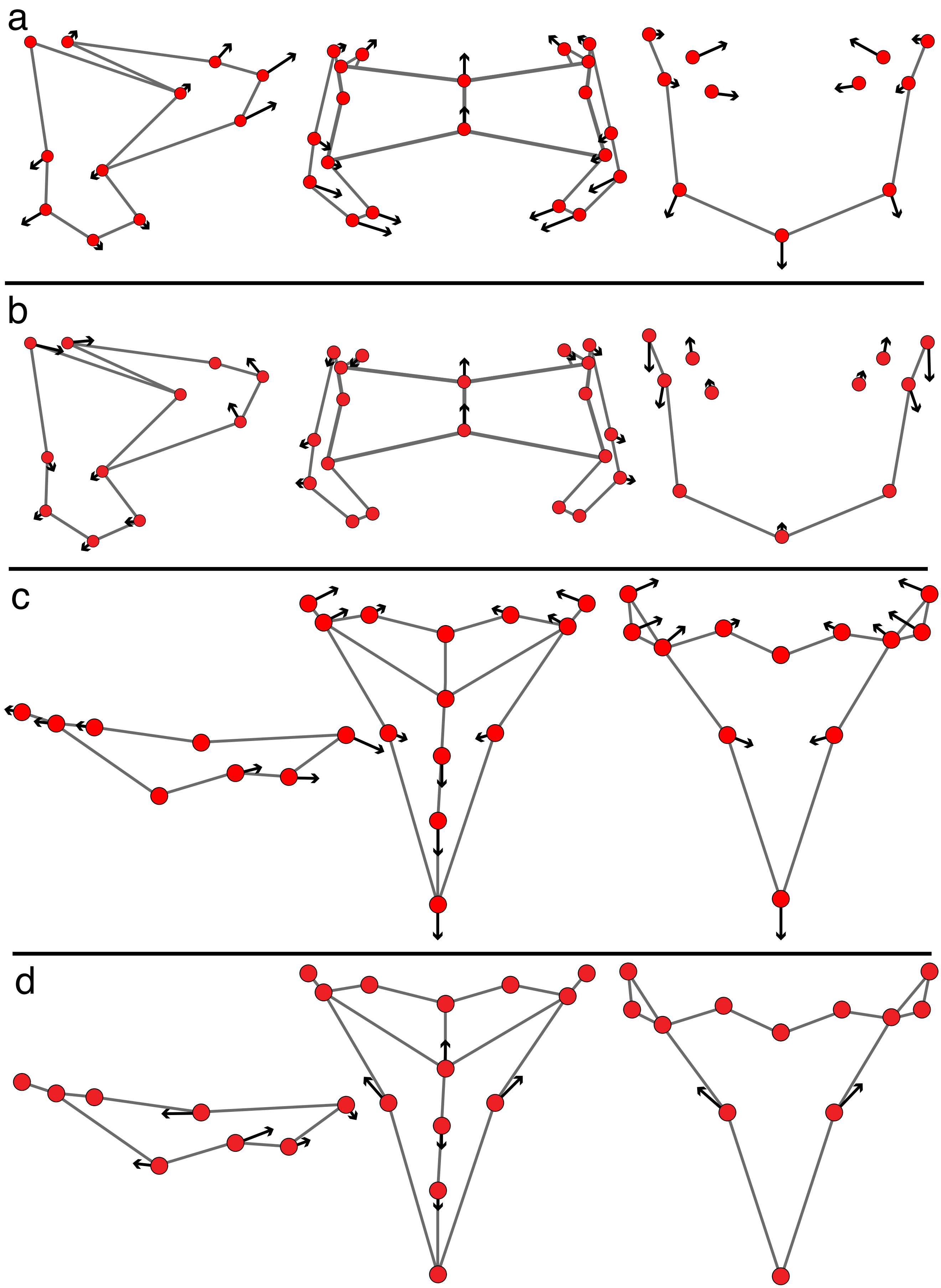
a
b
c
d

Supplementary Figure 5. Shape changes in oral and pharyngeal jaws for each principal component (PC). These four PCs were used as traits in the quantitative trait locus mapping analysis. a, PC1 lower oral jaw, arrows denote changes in overall length, width, and depth. b, PC2 lower oral jaw, arrows denote changes in aspects of the jaw processes, particularly the attachment site for the A2 muscle on the ascending arm of the mandible. Changing the size and shape of this process will have consequences for jaw closing mechanics. c, PC1 lower pharyngeal jaw, arrows denote changes in overall width, length, and wing process size. Note the size of the wing processes where the ADD5 muscle attaches. Also, changing the overall length will impact the attachment site for the PH muscle on the pharyngeal jaw keel processes. d, PC2 lower pharyngeal jaw, arrows denote changes in depth of the jaw, which would likely impact the size of the attachment sites for the PC-I and PC-E muscles, and thus their contraction strength.

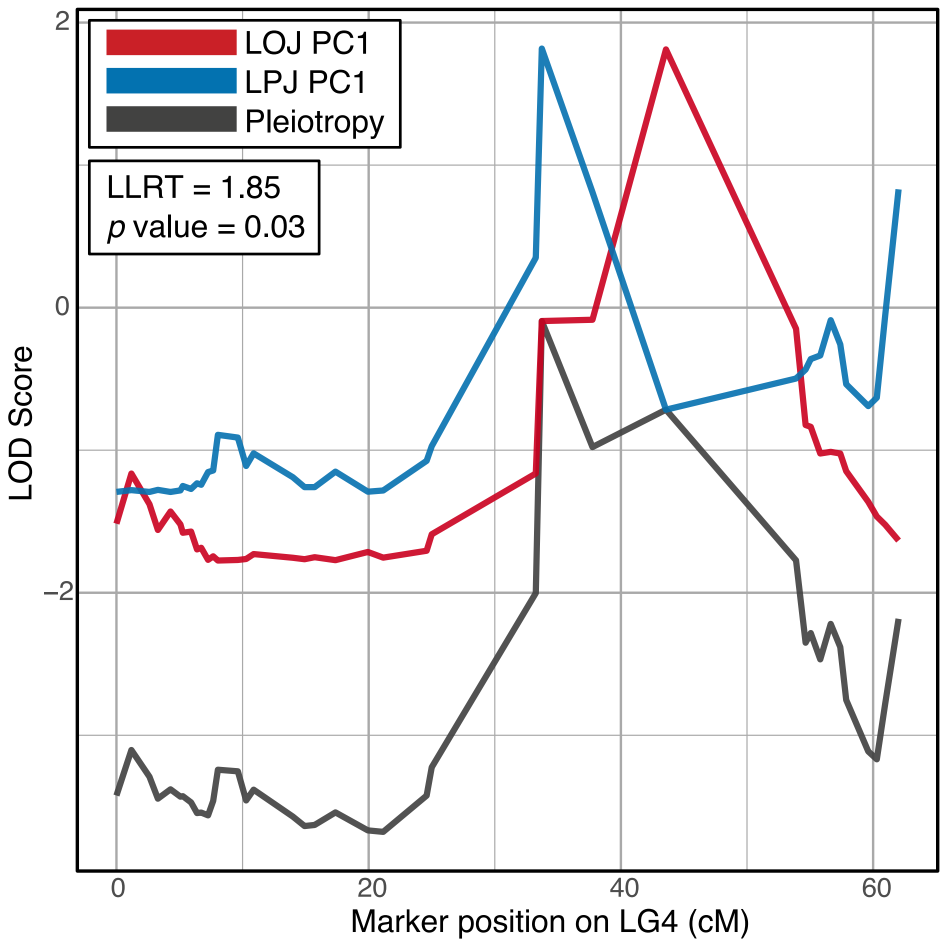

Supplementary Figure 6. Pleiotropy analysis spanning linkage group four. Plot depicts the likelihood of odds ratios for two traits and the pleiotropy trace to determine whether the oral jaw PC1 trait colocalizes to the same region as the pharyngeal jaw PC1 trait. Bootstrap permutation demonstrates that there is no evidence to suggest these traits on linkage group four are pleiotropic. The likelihood ratio test (LLRT) value and *p*-value are included.

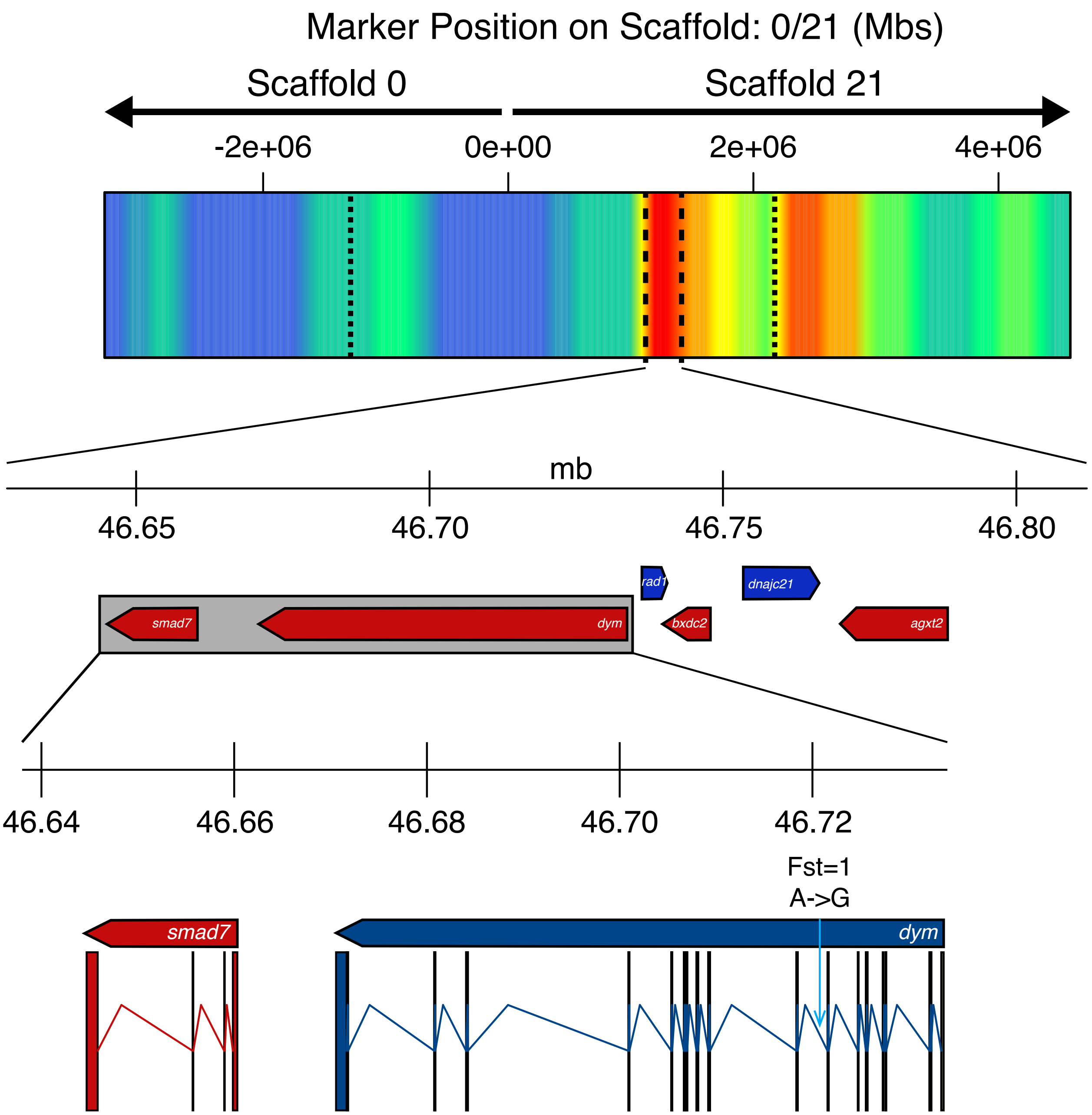

Supplementary Figure 7. Annotated schematic of the genomic region on LG7 that exhibited peak differences between hybrids of different genotypes. Top, Heatmap denoting an amalgamation of all three trait phenotypic effects following Z-score standardization, with warmer colors for the highest summed values of the traits, and cooler colors for the lowest. Middle, the schematic centers on those genes within the Bayes credible interval (dotted line) and also within the site of peak phenotypic effects that amounts to an ~200kb region (dashed line). Two genes within this region reflect major candidates for influencing bone shape in cichlid jaws: *smad7* and *dym*. Bottom, in *smad7* and *dym* we find a fully segregated F_{ST} in *dym* within intron 7 of 16, this SNP is located on LG7 at position 46720764.

Part III. Additional Citations

1. Rohlf, F. J. On applications of geometric morphometrics to studies of ontogeny and phylogeny. *Syst. Biol.* **47**, 147–167 (1998).
2. Adams, D. C., Collyer, M. L., Otarola-Castillo, E. & Sherratt, E. Geomorph: Software for geometric morphometric analyses. (2017).
3. Klingenberg, C. P., Barluenga, M. & Meyer, A. SHAPE ANALYSIS OF SYMMETRIC STRUCTURES: QUANTIFYING VARIATION AMONG INDIVIDUALS AND ASYMMETRY. *Evolution* **56**, 1909–1920 (2002).
4. Collyer, M. L., Sekora, D. J. & Adams, D. C. A method for analysis of phenotypic change for phenotypes described by high-dimensional data. *Heredity* **115**, 357–365 (2015).
5. Conith, M. R. *et al.* Genetic and developmental origins of a unique foraging adaptation in a Lake Malawi cichlid genus. *Proc. Natl Acad. Sci. USA* **115**, 7063–7068 (2018).
6. Ahmed, M. & Kim, D. R. pcr: an R package for quality assessment, analysis and testing of qPCR data. *PeerJ* **6**, e4473 (2018).
7. Liem, K. F. Evolutionary Strategies and Morphological Innovations: Cichlid Pharyngeal Jaws. *Syst. Zool.* **22**, 425–441 (1973).

Part IV. Citations for diet assignments in Supplementary Data 2

1. Arnegard, M. E., & Snoeks, J. New three-spotted cichlid species with hypertrophied lips (Teleostei: Cichlidae) from the deep waters of Lake Malaŵi/Nyasa, Africa. *Copeia* **3**, 705-717 (2001).
2. Conith, M. R., Conith, A. J. & Albertson, R. C. Evolution of a soft-tissue foraging adaptation in African cichlids: Roles for novelty, convergence, and constraint. *Evolution* **73**, 2072-2084 (2019).
3. Dominey, W. J., & Snyder, A. M. Kleptoparasitism of freshwater crabs by cichlid fishes endemic to Lake Barombi Mbo, Cameroon, West Africa. *Environ. Biol. Fishes* **22**, 155-160 (1988).
4. Genner, M. J., Turner, G. F., & Ngatunga, B. P. A guide to the tilapia fishes of Tanzania. 29pp. (2018).
5. Greenwood, P. H., The Haplochromine fishes of the East African lakes. Cornell University Press, 839pp. (1981).
6. Hata, H., Shibata, J., Omori, K. et al. Depth segregation and diet disparity revealed by stable isotope analyses in sympatric herbivorous cichlids in Lake Tanganyika. *Zoological Lett*. **1**, 15 (2015).
7. Hori, M., Yamaoka, K., & Takamura, K. Abundance and micro-distribution of cichlid fishes on a rocky shore of Lake Tanganyika. *Afr Stud Monogr*. **3**, 25-38 (1983).
8. Huber, R., van Staaden, M. J., Kaufman, L. S., & Liem, K. F. Microhabitat use, trophic patterns, and the evolution of brain structure in African cichlids. *Brain Behav Evol*. **50**, 167-182 (1997).
9. Kirchberger, P. C., Sefc, K. M., Sturmbauer, C., & Koblmüller, S. Evolutionary history of Lake Tanganyika's predatory deepwater cichlids. *Int J Evol Biol*. 716209 (2012).

10. Konan, Y. A., Ouattara, S., & Kone. T. Diet aspects of Thysochromis ansorgii (Cichlidae) in the Tanoe-Ehy swamp forest (Cote d'Ivoire). *Cybium* **38**, 261-266 (2014).

11. Konings, A. Tanganyika cichlids in their natural habitat, 3rd edition. Cichlid Press, El Paso, TX 408pp. (2015).

12. Konings, A. Malaŵi cichlids in their natural habitat, 5th edition. Cichlid Press, El Paso, TX 432pp. (2016).

13. Lamboj, A., The Cichlid Fishes of Western Africa. Birgit Schmettkamp Verlag, Bornheim, Germany. 255pp. (2004).

14. Li, S., Konings, A. F., & Stauffer, J. R., A Revision of the Pseudotropheus elongatus species group (Teleostei: Cichlidae) With Description of a New Genus and Seven New Species. *Zootaxa* **4168**, 353-381 (2016).

15. Martinez, C. M., McGee, M. D., Borstein, S. R. & Wainwright, P. C. Feeding ecology underlies the evolution of cichlid jaw mobility. *Evolution* **72**, 1645-1655. (2018).

16. McGee, M. D., Borstein, S. R., Meier, J. I. et al. The ecological and genomic basis of explosive adaptive radiation. *Nature* **586**, 75–79 (2020).

17. Muschick, M., Indermaur, A., & Salzburger, W. Convergent evolution with an adaptive radiation of cichlid fishes. *Curr Biol*. **22**, 2362-2368 (2012).

18. Navon, D., Male, I., Tetrault, E. R., Aaronson, B., Karlstrom, R. O., & Albertson, R. C. Hedgehog signaling is necessary and sufficient to mediate craniofacial plasticity in teleosts. *Proc. Natl Acad. Sci. USA* **117**, 19321-19327 (2020).

19. Reid, G. M., Threatened fishes of the world: Pungu maclareni (Trewavas, 1962) (Cichlidae). Environ. Biol. Fishes. **43**, 362 (1995).

20. Ribbink, A. J., Marsh, B. A., & Marsh, A. C., Ribbink, A. C., & Sharp, B. J. A preliminary survey of the cichlid fishes of rocky habitats in Lake Malawi. *South African J Zool*. **18**, 149–309 (1983).

21. Schwalbe, M. A. B., Bassett, D. K., & Webb, J. F. Feeding in the dark: lateral-line-mediated prey detection in the peacock cichlid Alonocara stuartgranti. *J Exp Biol*. **215**, 2060-2071 (2012).

22. Sidi Imorou, R., Adite, A., Sonon, S. P., Arame, H., & Adjibade, N. K. Trophic ecology of hemichromis fasciatus (pisces: cichlidae: perciformes) from opkara stream, oueme river, northern Benin: needs for species management and rational exploitation. *Asian J. Life Sci*. **7**, 46-61 (2019).

23. Stauffer, J. R. Jr., LoVullo, T. J., & McKaye, K. R. Three new sand-dwelling cichlids from Lake Malaŵi, Africa, with a discussion of the status of the genus Copadichormis (Teleostei: Cichlidae). *Copeia* **1993**, 1017-1027 (1993).

24. Stewart, D. J., & Roberts, T. R. A new species of dwarf cichlid fish with reversed sexual dichromatism from lac Mai-ndombe, Zaïre. *Copeia* **1984**, 82-86 (1984).

25. Stewart, T. A., & Albertson, R. C. Evolution of a unique predatory feeding apparatus: Functional anatomy, development, and a genetic locus for jaw laterality in Lake Tanganyika scale-eating cichlids. *BMC Biology* **8**, 8 (2010).

26. Sturmbauer C., Hainz U., Baric S., Verheyen E., & Salzburger W. Evolution of the tribe Tropheini from Lake Tanganyika: synchronized explosive speciation producing multiple evolutionary parallelism. *Hydrobiologia* **500**, 51-64 (2003).

27. Sturmbauer C., Meyer, A. Mitochondrial phylogeny of the endemic mouthbrooding lineages of cichlid fishes from Lake Tanganyika in Eastern Africa. *Mol Biol Evol*. **10**, 751-768 (1993).

28. Takahashi, R., Watanabe, K., Nishida, M. et al. Evolution of feeding specialization in Tanganyikan scale-eating cichlids: a molecular phylogenetic approach. *BMC Evol Biol*. **7**, 195 (2007).

29. Takamura, K. Interspecific relationships of aufwuchs-eating fishes in Lake Tanganyika.

Environ Biol Fish. **10**, 225-241 (1984).

30. Turner, G. F. Offshore Cichlids of Lake Malaŵi. Cichlid Press, Germany. 240pp. (1996).
