# Supplementary material for: The cichlid oral and pharyngeal jaws are evolutionarily and genetically coupled
Source: Nat Commun. 2021 Sep 16;12:5477. doi: 10.1038/s41467-021-25755-5 (PMC8445992; doi:10.1038/s41467-021-25755-5)
Supplement: Supplementary file 3 — Description of Additional Supplementary Files [file 41467_2021_25755_MOESM3_ESM.pdf]

Description of Additional Supplementary Files

Supplementary Data 1. All catalogue information for individuals present in the macroevolutionary component of the study that assessed cichlid jaw integration across lakes. Taxa present in this table were used to build Figure 2a.

Supplementary Data 2. Principal component scores output from all African cichlids for both jaws used in the macroevolutionary component of the study. Lake, clade, and diet assignments are also included. References for each diet assignment can be found in Part IV of the supplementary text. The *Aufwuchs* assignment refers to those species that feed by scraping filamentous algae from rocks and may sometimes eat small invertebrates in the process. The *Fish* assignment refers to those diets that contain almost exclusively fish prey. The *Omnivore* assignment refers to a diet that contains a mixture of algae, invertebrates, and small fish. The *Scale* assignment refers to those species that feed by ripping scales from the flank of other fish. The *Zoobenthos1* assignment refers to those diets that contain harder and/or more non-evasive invertebrates, typically requiring jaws specialized for power, while the *Zoobenthos2* assignment refers to those diets that contain softer and/or more evasive inverts, typically requiring jaws specialized for speed. The *Zooplankton* assignment refers to those species that feed primarily on small organisms from the water column.

Supplementary Data 2. Importance of the first five components extracted from a principal components analysis of the African cichlid mean shape data (plotted in Supplementary Figure 3).

Supplementary Data 4. All catalogue information for individuals present in the macroevolutionary component of the study that assessed cichlid jaw integration within the *Tropheops* species complex. Taxa present in this table were used to build Figure 2b.

Supplementary Data 5. All catalogue information for individuals present in the microevolutionary component of the study that assessed *Tropheops sp.* “red cheek” and

Labeotropheus fuelleborni jaw integration. Taxa present in this table were used to build Figure 3a-b.

Supplementary Data 6. Genetic map used to perform all quantitative trait locus (QTL) analyses. *Tropheops sp.* “red cheek” contributed the ‘A’ allele, while *Labeotropheus fuelleborni* contributed the ‘B’ allele at all marker positions.

Supplementary Data 7. Principal component scores output from all hybrids for both jaws. Further axes represented >10% of the variation and were omitted. Scores were used in the trait mapping component of the study.

Supplementary Data 8. QTL statistics output for all principal component traits scores. Significant intervals were plotted on Figure 4a.

Supplementary Data 9. Fine map that spans the entirety of linkage group seven.

Supplementary Data 10. Fine map that spans the entirety of the Bayesian credible interval and beyond made up of two scaffolds on linkage group seven.

Supplementary Data 11. Average phenotypic effect and allelic errors at each marker in the fine map for all three traits. Average phenotypic effect values (column AA.BB) were plotted in Figure 4d.

Supplementary Data 12. Average phenotypic effect at each marker in the fine map for all three traits. Average phenotypic effect values (column AA.BB) were plotted in Figure 4e. F_{ST} scores are also presented at each marker when available.

Supplementary Data 13. Testing efficiency of primers via quantitative PCR (qPCR) using the standard curve method. Raw output from qPCR that also shows the efficiency statistics at the bottom of the table.

Supplementary Data 14. Raw qPCR output for all taxa and tissues tested at 100ng/µL.

Supplementary Data 15. Relative expression values for both jaws for all taxa.

Supplementary Data 16. Detailed anatomical description of landmark placement. Landmarking number scheme matches the one used in Supplementary Figure 1a-b.

Supplementary Data 17. Allometric correction statistics for both jaws.

Supplementary Data 18. Symmetric correction statistics for both jaws.

Supplementary Data 19. Standard lengths for all individuals used in the qPCR experiment.
