# Supplementary material for: The cichlid oral and pharyngeal jaws are evolutionarily and genetically coupled
Source: Nat Commun. 2021 Sep 16;12:5477. doi: 10.1038/s41467-021-25755-5 (PMC8445992; doi:10.1038/s41467-021-25755-5)
Supplement: Supplementary file 5 — Reporting Summary [file 41467_2021_25755_MOESM5_ESM.pdf]

nature research

Corresponding author(s): Andrew J. Conith
R. Craig Albertson

Last updated by author(s): Aug 9, 2021

Reporting Summary

Nature Research wishes to improve the reproducibility of the work that we publish. This form provides structure for consistency and transparency in reporting. For further information on Nature Research policies, see our Editorial Policies and the Editorial Policy Checklist.

Statistics

For all statistical analyses, confirm that the following items are present in the figure legend, table legend, main text, or Methods section.

n/a	Confirmed	
☐	☒	The exact sample size (*n*) for each experimental group/condition, given as a discrete number and unit of measurement
☐	☒	A statement on whether measurements were taken from distinct samples or whether the same sample was measured repeatedly
☐	☒	The statistical test(s) used AND whether they are one- or two-sided<br>*Only common tests should be described solely by name; describe more complex techniques in the Methods section.*
☐	☒	A description of all covariates tested
☐	☒	A description of any assumptions or corrections, such as tests of normality and adjustment for multiple comparisons
☐	☒	A full description of the statistical parameters including central tendency (e.g. means) or other basic estimates (e.g. regression coefficient) AND variation (e.g. standard deviation) or associated estimates of uncertainty (e.g. confidence intervals)
☐	☒	For null hypothesis testing, the test statistic (e.g. *F*, *t*, *r*) with confidence intervals, effect sizes, degrees of freedom and *P* value noted<br>*Give P values as exact values whenever suitable.*
☐	☒	For Bayesian analysis, information on the choice of priors and Markov chain Monte Carlo settings
☒	☐	For hierarchical and complex designs, identification of the appropriate level for tests and full reporting of outcomes
☒	☐	Estimates of effect sizes (e.g. Cohen's *d*, Pearson's *r*), indicating how they were calculated

Our web collection on statistics for biologists contains articles on many of the points above.

Software and code

Policy information about availability of computer code

Data collection	Micro-CT data all collected using a X-Tek HMXST 225 (Nikon Corporation). Scans were processed using Mimics v.19 (Materialise NV), before exporting the 3D models to Geomagic 2014 (v.1.0 3D Systems). All landmarking performed using Landmark Editor (v.3.0).
Data analysis	All statistical analyses were conducted using the R statistical programming language version 4.0.1.<br>Analyses utilized routines built into two major R packages: Geomorph version 3.3.1 and r/qtl version 1.46-2.<br>RAD-seq data was output in a VCF file that was quality checked using VCFtools version 0.1.16.<br>Scripts and raw data for all analyses can be viewed on GitHub at github.com/andrewjohnconith/cichlid_OJPJ

For manuscripts utilizing custom algorithms or software that are central to the research but not yet described in published literature, software must be made available to editors and reviewers. We strongly encourage code deposition in a community repository (e.g. GitHub). See the Nature Research guidelines for submitting code & software for further information.

Data

Policy information about availability of data

All manuscripts must include a data availability statement. This statement should provide the following information, where applicable:

- Accession codes, unique identifiers, or web links for publicly available datasets
- A list of figures that have associated raw data
- A description of any restrictions on data availability

Additional data and tables can be found in the supplementary information.
Supplementary data includes all genomic maps used in this analysis. Additional cichlid taxa were added to the dataset from the online repository morphosource.org.

Field-specific reporting

Please select the one below that is the best fit for your research. If you are not sure, read the appropriate sections before making your selection.

☐ Life sciences ☐ Behavioural & social sciences ☒ Ecological, evolutionary & environmental sciences

For a reference copy of the document with all sections, see nature.com/documents/nr-reporting-summary-flat.pdf

Ecological, evolutionary & environmental sciences study design

All studies must disclose on these points even when the disclosure is negative.

Study description	This study assessed the evolutionary and genetic association (or lack thereof) of the cichlid oral and pharyngeal jaws. These features reflect two major components of the feeding system that can operate independently, and we assess if they also evolved independently and shape variation is controlled by independent genetic regions.<br>We assess (de)coupling in jaw shape across a macroevolutionary and microevolutionary sample using multivariate methods (geometric morphometrics). We then use the same methods to extract shape information from a cichlid hybrid mapping population and perform quantitative trait loci analysis on the shape axes. We identify a handful of candidate genes and perform quantitative PCR to determine differences in expression levels of our different genes.<br>The methods applied in this study do not follow a typical design structure (e.g. factorial, nested, hierarchical).
Research sample	African cichlids from the rift lake valley - all cichlids in the evolutionary component of the study come from three lakes: Lake Victoria, Lake Malawi, and Lake Tanganyika. The majority of cichlids used in this study originated in Lake Malawi. This study focused on Lake Malawi as the cichlid radiation in this lake was rapid and produced large numbers of trophically diverse members that would be critical in assessing how oral and pharyngeal jaw shape is (de)coupled in trophic generalists or specialists and how it may change with diet. Cichlids used in the shape analyses were adults, and those used in the molecular component were juveniles (inc. both sexes).
Sampling strategy	Macroevolutionary study.<br>Sample contains representative cichlids from all three rift lakes, and includes examples of trophic specialists that typically exhibit more extreme morphologies that could define the limits of our morphological axes, and also representatives from almost all the available diets available to cichlids in these lakes.<br>Microevolutionary study.<br>Sample contains almost all known Tropheops species from across the southern part of Lake Malawi and also includes the same species from different localities.<br>Hybrid Mapping<br>On average, most cichlid mapping studies contain 200-300 individuals to obtain high confidence in QTL peaks, our study contained more than 400 hybrid individuals from an F5 population to drastically improve the power and resolution of any prospective QTL peaks.<br>Expression studies<br>We used three different cichlid species that are well characterized in the field (Tropheops, Maylandia, and Labeotropheus), and assayed 6-8 individuals for each, at least 3 individuals are typically used in expression analyses.
Data collection	All data was collected by a single author (AJC) to eliminate interobserver error.
Timing and spatial scale	Most data were collected between 2018 and 2020. Tropheops species were collected from a single trip to Lake Malawi in 2001.
Data exclusions	No data excluded from the analysis.
Reproducibility	We have provided all data used in the study in online repositories for replication. Where possible we obtained measurements from multiple individuals of the same species and used species means in all phylogenetic comparative analyses. All species with multiple individuals exhibited low variation relative to across species differences.
Randomization	Randomization is not appropriate for the analyses in this study. Cichlids were grouped by lake, diet, or phylogenetic position.
Blinding	It was not possible to blind authors during tissue collection for the expression analysis, nor is it possible to blind the phenotyping component as jaw shapes are distinct. Phenotyping of hybrid population occurred prior to RAD-seq genotyping such that authors were blind to the genetic background of each hybrid.

Did the study involve field work? ☒ Yes ☐ No

Field work, collection and transport

Field conditions	Fish were collected from the field in July 2001. Conditions were standard (i.e., not anomalous) for that time of year - dry with day time temperatures around 23 degrees C.
Location	Fish were collected from the southern part of Lake Malawi, at depths ranging from 1 to 20 meters.
Access & import/export	Fish were collected under permits issued by the government of Malawi in 2001, including the departments of Fisheries and National Parks. This was 20 years ago. The PI was a graduate student at the time, and hence the permits were issued to his advisor.

Disturbance	Fish were collected from the water column with monofilament nets while SCUBA diving. Fish were collected one at a time to minimize disturbance of the surrounding community.

Reporting for specific materials, systems and methods

We require information from authors about some types of materials, experimental systems and methods used in many studies. Here, indicate whether each material, system or method listed is relevant to your study. If you are not sure if a list item applies to your research, read the appropriate section before selecting a response.

Materials & experimental systems

n/a	Involved in the study
☒	☐ Antibodies
☒	☐ Eukaryotic cell lines
☒	☐ Palaeontology and archaeology
☐	☒ Animals and other organisms
☒	☐ Human research participants
☒	☐ Clinical data
☒	☐ Dual use research of concern

Methods

n/a	Involved in the study
☒	☐ ChIP-seq
☒	☐ Flow cytometry
☒	☐ MRI-based neuroimaging

Animals and other organisms

Policy information about studies involving animals; ARRIVE guidelines recommended for reporting animal research

Laboratory animals	Cichlids. Three species: Tropheops kumara, Labeotropheus fuelleborni, Maylandia callainos. Hybrid cichlid population: Labeotropheus fuelleborni X Tropheops sp. "red cheek." All cichlids were juveniles.
Wild animals	All work was performed in compliance with the Institutional Animal Care and Use Committee at UMass Amherst (#2018-0094 to RCA). Collection permits were issued though the University of Malawi and the Malawi government to collect adult Tropheops species (both sexes) via nets. Fish were transported in ethanol soaked cloth and cleaned using dermestid beetles. MS222 was used as an anesthetic and euthanasia agent in fish ready for µCT scanning.
Field-collected samples	All Tropheops species were caught in Lake Malawi, euthanized, and stored in ethanol prior to transport.
Ethics oversight	All work was performed in compliance with the Institutional Animal Care and Use Committee at UMass Amherst (#2018-0094 to RCA). Collection permits were issued though the University of Malawi and the Malawi government.

Note that full information on the approval of the study protocol must also be provided in the manuscript.
